# An Improved Synthesis of 1,2-Diarylethanols under Conventional Heating and Ultrasound Irradiation

**DOI:** 10.3390/molecules170910708

**Published:** 2012-09-07

**Authors:** Dong-Mei Gao, Wei-Li Ma, Tian-Rui Li, Liang-Zhu Huang, Zhen-Ting Du

**Affiliations:** College of Science, Northwest A&F University, Yangling 712100, Shaanxi, China

**Keywords:** 1,2-diarylethanols, ultrasonic-assisted organic synthesis, 2-nitrotoulene

## Abstract

A simple and efficient synthesis of 1,2-diarylethanols has been developed. The procedure involved the reaction between a variety of toluene derivatives and aryl aldehydes under conventional heating and ultrasound irradiation. This procedure possesses several advantages such as operational simplicity, high yield, safety and environment benignancy. Ultrasound was proved to be very helpful to the reaction, markedly improving the yield and the reaction rate.

## 1. Introduction

**Figure 1:**
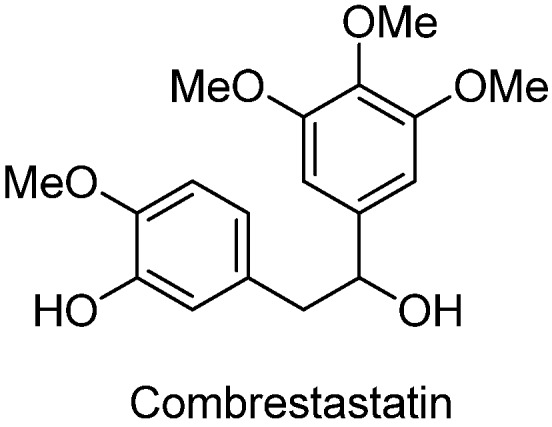
Representative structures of 1,2-diarylethanols.

1,2-diarylethanols are important intermediates in organic synthesis and can be used in a variety of natural and biological products such as the well-known resveratrol and derivatives [1], combrestastatins [2], isocombretastatins [3]. As a representative, combrestastatin (Figure 1), which was separated from African plants, inhibits growth of the murine P388 lymphocytic leukemia cell line (PS system) significantly, reverses the differentiation of AC glioma cells into astrocytes and inhibits tubulin polymerization [4,5].

The traditional formation of 1,2-diarylethanols need highly reactive agents such as titanium [6], the Barbier reaction or Grignard reagents. The method suffered some drawbacks such as harsh reaction conditions (high temperature and basic conditions), unpredictable yields, use of expensive metals and limited applicability. Murata reported a method for the synthesis of 1,2-diarylethanols in only 44% yield by refluxing a mixture of 2-nitrotoulene, sodium ethoxide and aldehyde for 2 days [7]. Therefore, there is still a need for general methods for the preparation of 1,2-diarylethanols.

Ultrasound can accelerate chemical reactions to provide improved yields, shortened reaction times and increased selectivity, therefore ultrasound irradiation has been recognized as an efficient technique in organic synthesis over the last two decades [8,9,10,11,12,13,14]. Typically, ultrasound works by the phenomenon of cavitation; which involves the growth, oscillation, and collapse of bubbles under the action of an acoustic field. There are three different theories about cavitation: the hotspot, the electrical and the plasma theory; and the most popular one is the hot spot theory. It has been experimentally shown that, the cavitational collapse creates drastic conditions inside the medium for an extremely short time and temperatures of 2,000–5,000 K, pressures up to 1,800 atmosphere pressure. Inside the collapsing cavity have been produced under sonic conditions [14,15]. The collapse causes a couple of strong physical effects outside the bubble such as-shear forces, jets and shock waves. These cavitation-induced effects can cause physical, chemical, and biological transformations more effectively. Thus, ultrasound has found applications in synthetic chemistry, in materials science, in life sciences, as well as in medicinal chemistry [16]. 

As mentioned above, we were interested in a practical technique for the synthesis of 1,2-diarylethanols. To best of our knowledge, the ultrasound-assisted methodology has not yet been explored in the formation of 1,2-diarylethanols. Herein, we wish to report our studies toward the coupling of 2-nitrotoluenes with aldehydes in the presence of sodium ethoxide as the base to produce diverse 1,2-diarylethanols under conventional and ultrasound irradiation conditions. 

## 2. Results and Discussion

To begin with, we intend to screen the most suitable base and solvent for this model reaction in which 2-nitrotoulene and benzaldehyde were chosen as substrates under ultrasound irradiation. At 40 °C, K_2_CO_3_ and NaOH give low yields in ethanol, however, when organic bases such as DBU and NaOEt were used, the yields were improved slightly. When DMSO was used as solvent, although the TLC showed promising results, the distillation workup would produce byproducts due to elimination. Eventually, a mixed solvent (EtOH–DMSO = 6:1) was used to overcome this drawback; apparently, it is helpful to improve the yield (Table 1, entries 6–9). Finally, we investigated the influence of temperature (such as at 40 °C, 50 °C, 60 °C and 70 °C) on the reaction, and we found that the reaction temperature played a key role. For example, a higher temperature may give better yield (Table 1, entries 6–7), but if the temperature was increased to 60 °C, the side reaction would prevail. Unfortunately, this side reaction affected the yield immensely at 70 °C. Finally, we set up the optimized reaction conditions that are NaOEt as base, the mixed solvent (EtOH–DMSO = 6:1), at 50 °C (Table 1, entry 7).

**Table 1:** Screening the solvent, base and temperature for synthesis of 2-(2-nitro-phenyl)-1-phenylethanol where the molar ratio of 2-nitrotoulene:benzaldehyde is 2:1.

Entry	Base	Solvent	Temperature	Time	Yield (%) ^a^
			°C	h	
1	K_2_CO_3_	EtOH	40	5	0
2	NaOH	EtOH	40	5	5
3	DBU	EtOH	40	5	8
4	NaOEt	EtOH	40	5	22
5	NaOEt	DMSO	40	5	44
6	NaOEt	EtOH:DMSO (6:1)	40	8	53
7	NaOEt	EtOH:DMSO (6:1)	50	8	62
8	NaOEt	EtOH:DMSO (6:1)	60	8	56
9	NaOEt	EtOH:DMSO (6:1)	70	8	28 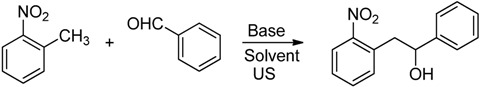

^a^ Isolated yields.

Under these optimized reaction conditions, we next examined the scope of ultrasound irradiation-promoted synthesis of 2-(2-nitrophenyl)-1-phenylethanol using 2-nitrotoulenes and benzaldehydes. The results are summarized in Table 2. 2-Nitrotoulene and 2,4-dinitrotoulene and a wide range of aryl aldehydes (Table 2), bearing chloro (Table 2, entries d, e and j), bromo (entry c) and methoxy group (entry b) substituents were subjected to this protocol to give the corresponding 1,2-diarylethanols in good yields. Meanwhile, the reaction time was reduced significantly compared with the conventional thermal method (2 days). For example, 2-nitrotoulene was coupled with benzaldehyde to provide the expected 1-phenyl-2-(2'-nitro)phenyl ethanol in 62% yield after only 8 h under sonication at 50 °C. It should be pointed out that two activitated groups didn't show a promising effects in this reaction (entries e, j), for instance, 2,4-dinitrotoulene reacted with 2-chlorobenzaldehyde in low yield (entry j, 35%) compared with 2-nitrotoulene (entry e, 58%). Moreover, as we all know furaldehyde is unstable under harsh conditions, but it could provide the corresponding 1,2-diarylethanol through our protocol in 53% yield. All the products in our reactions listed in Table 2 were easily characterized on the basis of physical and spectral data and also by comparison with authentic samples.

**Table 2:** Ultrasound-assisted synthesis of 1,2-arylethanols using nitrotoluenes and arylaldehydes.

Entry	Compound 1	Compound 2	Product 3	Time (h)	Yield (%) ^a^
a	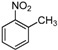	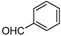	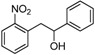	8	62
b	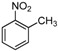	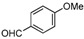	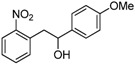	8	65
c	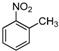	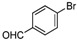	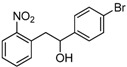	12	60
d	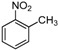	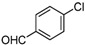	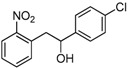	12	49
e	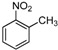	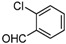	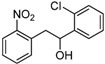	12	58
f	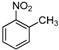	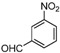	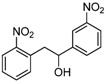	12	46
g	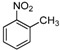	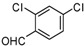	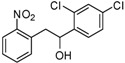	8	44
h	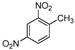	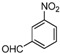	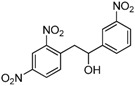	12	45
i	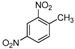	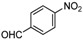	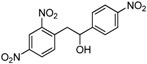	8	44
j	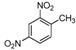	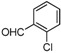	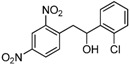	8	35
k	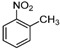	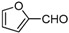	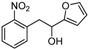	8	53 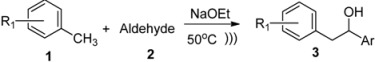

^a^: isolated yield.

## 3. Experimental

### 3.1. General

Sonication was performed in a Ningbo SB-3200DT ultrasonic cleaner (Xi’an, China) with a frequency of 40 KHz and an output power of 180 W. the temperature of the water was maintained through an outside cooling loop system. The materials were used as purchased if not noted otherwise and DMSO was used directly without any additional purification. Melting points were uncorrected. ^1^H-NMR and ^13^C-NMR spectra were recorded on a Bruker Avance DMX 500 MHz instrument (Xi’an, China) using TMS as internal standard and CDCl_3_ as solvent. MS were carried out with a Thermo DSQ GC/MS instrument.

### 3.2. General Procedure

The mixture of the appropriate nitrotoluenes (**1**, 10 mmol), the aldehydes (**2**, 5 mmol), and freshly prepared NaOEt (20 mmol) in DMSO (6 mL) in EtOH (36 mL) in a shaken flask was reacted using sonication at 50 °C under argon. The reaction progress was monitored by TLC, after completion, the reaction mixtures were poured into ice water, acidified to pH 2–3, and extracted with ethyl acetate (3 × 30 mL). The combined organic phase was washed by brine (40 mL), dried over anhydrous MgSO_4_, concentrated in *vacuo* and purified by preparative column chromatography to give the pure 1,2-diaryl-ethanols. All compounds were characterized by ^1^H-NMR, MS and m.p. The yields are listed in Table 2.

*1-Phenyl-2-(2-nitrophenyl)ethanol* (**3a**). White solid, m.p. 74–75 °C, Lit [17] 70–72 °C; ^1^H-NMR (CDCl_3_): 3.23 (dd, 1H, *J* = 4.7 Hz, *J* = 13.5 Hz), 3.39 (dd, 1H, *J* = 3.8 Hz, *J* = 13.6 Hz), 5.03 (dd, 1H, *J* = 3.8 Hz, *J* = 12.1 Hz), 7.28–7.40 (m, 7H), 7.51 (t, 1H, *J* = 7.5 Hz), 7.94 (d, 1H, *J* = 8.2 Hz); ^13^C-NMR (CDCl_3_): 42.8, 74.3, 124.8, 125.7, 127.7, 127.9, 128.6, 132.7, 133.4, 133.6, 143.8, 149.9. 

*1-(4-Methoxyphenyl)-2-(2-nitrophenyl)ethanol* (**3b**). Orange solid, m.p. 91–92 °C; ^1^H-NMR (CDCl_3_): 3.23 (dd, 1H, *J* = 9.0 Hz, *J* = 14.0 Hz), 3.32 (dd, 1H, *J* = 4.0 Hz, *J* = 13.5 Hz), 3.80 (s, 3H), 4.96 (dd, 1H, *J* = 4.0 Hz, *J* = 8.0 Hz), 6.88 (d, 2H, *J* = 8.6 Hz), 7.28–7.31 (m, 3H), 7.38 (t, 1H, *J* = 5.1 Hz), 7.50 (t, 1H, *J* = 4.7 Hz), 7.92 (d, 1H, *J* = 7.9 Hz); ^13^C-NMR (CDCl_3_): 42.8, 55.3, 73.9, 113.9, 124.8, 126.9, 127.6, 132.7, 133.5, 133.6, 136.0, 149.9, 159.2.

*1-(4-Bromophenyl)-2-(2-nitrophenyl)ethanol* (**3c**). White solid, m.p. 71–72 °C; ^1^H-NMR (CDCl_3_): 3.17 (dd, 1H, *J* = 8.8 Hz, *J* = 13.6 Hz), 3.35 (dd, 1H, *J* = 4.0 Hz, *J* = 13.6 Hz), 5.01 (dd, 1H, *J* = 4.3 Hz, *J* = 8.7 Hz), 7.28 (d, 1H, *J* = 8.3 Hz), 7.30 (d, 1H, *J* = 8.7 Hz), 7.41 (t, 2H, *J* = 7.7 Hz), 7.48 (d, 1H, *J* = 8.3 Hz), 7.52 (t, 2H, *J* = 7.5 Hz), 7.96 (d, 1H, *J* = 8.1 Hz); ^13^C-NMR (CDCl_3_): 42.9, 73.7, 121.6, 124.9, 127.4, 127.9, 131.6, 132.9, 133.0, 133.6, 142.8, 149.8.

*1-(4-Chlorophenyl)-2-(2-nitrophenyl)ethanol* (**3d**). White solid, m.p. 89–91 °C; ^1^H-NMR (CDCl_3_): 3.17 (dd, 1H, *J* = 9.1 Hz, *J* = 12.8 Hz), 3.34 (dd, 1H, *J* = 2.0 Hz, *J* = 13.5 Hz), 3.47 (s, 1H), 5.03 (dd, 1H, *J* = 3.5 Hz, *J* = 8.5 Hz), 7.29–7.33 (m, 5H), 7.41 (t, 1H, *J* = 7.6 Hz), 7.52 (t, 1H, *J* = 7.3 Hz), 7.96 (d, 1H, *J* = 8.1 Hz); ^13^C-NMR (CDCl_3_): 43.0, 73.6, 124.9, 127.1, 127.1, 128.7, 132.9, 133.0, 133.5, 133.6, 142.3, 149.8.

*1-(2-Chlorophenyl)-2-(2-nitrophenyl)ethanol* (**3e**). Pale yellow solid, m.p. 93–94 °C, Lit [18] 59–60 °C; ^1^H-NMR (CDCl_3_): 3.35 (d, 2H, *J* = 5.9 Hz), 5.40 (t, 1H, *J* = 5.8 Hz), 7.23 (d, 1H, *J* = 7.6 Hz), 7.27 (t, 1H, *J* = 6.5 Hz), 7.31 (d, 1H, *J* = 6.5 Hz), 7.38 (t, 2H, *J* = 7.7 Hz), 7.50 (t, 1H, *J* = 9.0 Hz), 7.53 (d, 2H, *J* = 7.0 Hz), 7.87 (d, 1H, *J* = 8.1 Hz); ^13^C-NMR (CDCl_3_): 39.9, 71.3, 124.6, 127.3, 127.3, 127.7, 128.9, 129.4, 131.7, 132.7, 132.7, 133.0, 140.8, 150.5.

*1-(3-Nitrophenyl)-2-(2-nitrophenyl)ethanol* (**3f**). White solid, m.p. 98–99 °C; ^1^H-NMR (CDCl_3_): 3.17 (dd, 1H, *J* = 8.9 Hz, *J* = 13.6 Hz), 3.44 (dd, 1H, *J* = 3.6 Hz, *J* = 13.6 Hz), 5.19 (dd, 1H, *J* = 3.1 Hz, *J* = 8.7 Hz), 7.34 (d, 1H, *J* = 7.6 Hz), 7.45 (t, 1H, *J* = 7.8 Hz), 7.55 (q, 2H, *J* = 7.7 Hz), 7.75 (d, 1H, *J* = 7.6 Hz), 7.99 (d, 1H, *J* = 8.2 Hz), 8.15 (d, 1H, *J* = 8.1 Hz), 8.30 (s, 1H); ^13^C-NMR (CDCl_3_): 43.1, 73.2, 120.7, 122.7, 125.1, 128.2, 129.5, 132.0, 132.5, 133.1, 133.7, 146.0, 148.4, 149.7.

*1-(2,4-Dichlorophenyl)-2-(2-nitrophenyl)ethanol* (**3g**). Pale yellow solid, m.p. 88–89 °C; ^1^H-NMR (CDCl_3_): 3.29 (dd, 1H, *J* = 6.1 Hz, *J* = 13.9 Hz), 3.34 (dd, 1H, *J* = 4.8 Hz, *J* = 13.9 Hz), 5.37 (dd, 1H, *J* = 5.0 Hz, *J* = 7.5 Hz), 7.25–7.34 (m. 3H), 7.40 (t, 1H, *J* = 7.7 Hz), 7.47 (d, 1H, *J* = 8.5 Hz), 7.51 (t, 1H, *J* = 7.5 Hz), 7.88 (d, 1H, *J* = 8.1 Hz); ^13^C-NMR (CDCl_3_): 39.8, 70.8, 124.7, 127.6, 127.9, 128.4, 129.1, 132.2, 132.3, 132.8, 133.0, 133.9, 139.5, 150.5.

*1-(3-Nitrophenyl)-2-(2,4-dinitrophenyl)ethanol* (**3h**). Yellow solid, m.p. 116–117 °C; ^1^H-NMR (CDCl_3_): 3.30 (dd, 1H, *J* = 9.4 Hz, *J* = 12.7 Hz), 3.54 (d, 1H, *J* = 12.9 Hz), 5.23 (dd, 1H, *J* = 4.1 Hz, *J* = 8.3 Hz), 7.58 (t, 1H, *J* = 7.7 Hz), 7.67 (d, 1H, *J* = 8.4 Hz), 7.78 (d, 1H, *J* = 7.3 Hz), 8.18 (d, 1H, *J* = 7.6 Hz), 8.31 (s, 1H); 8.40 (d, 1H, *J* = 7.9 Hz), 8.82 (s, 1H); ^13^C-NMR (CDCl_3_): 42.6, 72.8, 120.4, 120.5, 123.1, 126.8, 129.8, 131.8, 135.3, 139.6, 145.4, 147.0, 148.5, 149.7.

*1-(4-Nitrophenyl)-2-(2,4-dinitrophenyl)ethanol* (**3i**). Yellow solid, m.p. 159–160 °C; ^1^H-NMR (CDCl_3_): 3.28 (dd, 1H, *J* = 8.8 Hz, *J* = 13.1 Hz), 3.52 (dd, 1H, *J* = 3.2 Hz, *J* = 13.4 Hz), 5.12 (dd, 1H, *J* = 3.1 Hz, *J* = 8.2 Hz), 7.61 (d, 2H, *J* = 8.1 Hz), 7.68 (d, 1H, *J* = 8.3 Hz), 8.23 (d, 2H, *J* = 8.2 Hz), 8.40 (d, 1H, *J* = 8.1 Hz), 8.81 (s, 1H); ^13^C-NMR (CDCl_3_): 44.6, 74.1, 122.0, 122.2, 125.7, 128.6, 137.4, 142.2,148.8, 149.3, 151.7, 153.7.

*1-(2-Chlorophenyl)-2-(2,4-dinitrophenyl)ethanol* (**3j**). Yellow solid, m.p. 134–135 °C; ^1^H-NMR (CDCl_3_): 3.28 (dd, 1H, *J* = 8.6 Hz, *J* = 13.6 Hz), 3.46 (dd, 1H, *J* = 7.5 Hz, *J* = 13.4 Hz), 5.00 (dd, 1H, *J* = 7.4 Hz, *J* = 13.4 Hz), 7.28–7.51 (m, 3H), 7.50 (d, 1H, *J* = 7.6 Hz), 7.57 (d, 1H, *J* = 8.2 Hz), 8.35 (d, 1H, *J* = 8.1 Hz), 8.80 (s, 1H); ^13^C-NMR (CDCl_3_): 42.6, 72.9, 120.2, 122.0, 126.5, 127.3, 131.8, 135.2, 140.2, 142.4, 146.9, 149.9.

*1-(Furan-2-yl)-2-(2-nitrophenyl)ethanol* (**3k**). colorless liquid; ^1^H-NMR (CDCl_3_): 3.41 (dd, 1H, *J* = 8.2 Hz, *J* = 13.6 Hz), 3.46 (dd, 1H, *J* = 5.2 Hz, *J* = 13.6 Hz), 5.03 (dd, 1H, *J* = 5.0 Hz, *J* = 8.6 Hz), 6.22 (d, 1H, *J* = 3.1 Hz), 6.32 (dd, 1H, *J* = 1.7 Hz, *J* = 3.0 Hz), 7.31 (d, 1H, *J* = 7.6 Hz), 7.36–7.52 (m, 3H), 7.92 (d, 1H, *J* = 8.2 Hz); ^13^C-NMR (CDCl_3_): 39.0, 67.9, 106.6, 110.3, 124.8, 127.8, 132.7, 132.9, 133.3, 142.2, 149.9, 155.4.

## 4. Conclusions

In summary, we have applied ultrasound methodology to promote the synthesis of 1,2-diarylethanols in moderate to good yields in the presence of sodium ethoxide. The merits of this protocol such as easy operation and workup, and good to moderate yields make it an attractive approach to such kinds of compounds.
